# Viral load suppression and acquired HIV drug resistance in adults receiving antiretroviral therapy in Viet Nam: results from a nationally representative survey

**DOI:** 10.5365/wpsar.2018.9.1.008

**Published:** 2018-08-20

**Authors:** Vu Quoc Dat, Bui Duc Duong, Do Thi Nhan, Nguyen Huu Hai, Nguyen Thi Lan Anh, Huynh Hoang Khanh Thu, Tran Ton, Luong Que Anh, Nguyen Tuan Nghia, Nguyen Vu Thuong, Khuu Van Nghia, Tran Thi Minh Tam, Tran Phuc Hau, Nguyen Duy Phuc, Vu Xuan Thinh, Nguyen Tran Hien, Truong Thi Xuan Lien, Silvia Bertagnolio, Nguyen Thi Thuy Van, Masaya Kato

**Affiliations:** a Department of Infectious Diseases, Hanoi Medical University, Hanoi, Vietnam.; b Vietnam Authority of HIV/AIDS Control, Hanoi, Vietnam.; c National Institute of Hygiene Epidemiology (NIHE), Hanoi, Vietnam.; d Pasteur Institute of Ho Chi Minh City, Ho Chi Minh City, Vietnam.; e World Health Organization, Geneva, Switzerland.; f World Health Organization Viet Nam Country Office, Hanoi, Vietnam.

## Abstract

**Objective:**

The purpose of this survey was to estimate the prevalence of viral load (VL) suppression and emergence of HIV drug resistance (HIVDR) among individuals receiving antiretroviral therapy (ART) for 36 months or longer in Viet Nam using a nationally representative sampling method.

**Methods:**

The survey was conducted between May and August 2014 using a two-stage cluster design. Sixteen ART clinics were selected using probability proportional to proxy size sampling, and patients receiving ART for at least 36 months were consecutively enrolled. Epidemiological information and blood specimens were collected for HIV-1 VL and HIVDR testing; HIVDR was defined by the Stanford University HIVDR algorithm.

**Results:**

Overall, 365 eligible individuals were recruited with a mean age of 38.2 years; 68.4% were men. The mean time on ART was 75.5 months (95% confidence interval [CI]: 69.0–81.9 months), and 93.7% of the patients were receiving non-nucleoside reverse transcriptase inhibitor-based regimens. Of the 365 individuals, 345 (95.1%, 95% CI: 92.3–96.9%) had VL below 1000 copies/mL and 19 (4.6%, 95% CI: 2.8-–7.5) had HIVDR mutations.

**Discussion:**

Our nationally representative survey found a high level of VL suppression and a low prevalence of HIVDR among individuals who received ART for at least 36 months in Viet Nam. Continued surveillance for HIVDR is important for evaluating and improving HIV programs.

## Introduction

There were an estimated 250 000 people living with HIV in Viet Nam in 2016. (*1*) The HIV epidemic in Viet Nam remains concentrated primarily among people who inject drugs (PWID), female sex workers (FSW) and men who have sex with men (MSM). According to HIV sentinel surveillance, the HIV prevalence was 11.0% in PWID, 2.7% in FSW and 8.2% in MSM in 2016. (*2*)

Antiretroviral therapy (ART) was first introduced in Viet Nam in the mid-1990s and has been rapidly scaled up since 2005, with a total of 115 927 people receiving ART at the end of 2016. (*2*, *3*) However, the prevalence of viral load (VL) suppression and HIV drug resistance (HIVDR) patterns at the national scale were unknown. There have been several HIVDR surveys undertaken in Viet Nam in the past decade. However, no study provided a nationally representative estimate of VL suppression and acquired HIV drug resistance (ADR).

Prior to 2011, the World Health Organization (WHO) recommended the prospective cohort studies of patients in conveniently selected sentinel sites to assess the emergence of ADR. (*4*) However, considerable financial and human resources are required for the recruitment and maintenance of a prospective cohort. Moreover, due to the nature of the survey design, the delay between the initiation of the survey and the dissemination of the results was longer than 24 months, preventing the use of this information for timely public health action. To address these implementation challenges and to ensure findings fully reflect the situation in the national programme, WHO developed a new survey method using a cross-sectional approach to estimate the level of VL suppression and ADR using a nationally representative sample of people receiving ART in the country. (*5*) The survey can be implemented quickly and the results are nationally representative; thus, it has greater potential to inform the public health response in timely manner.

In 2014, Viet Nam became one of the first countries in the world to conduct an ADR survey using the new WHO guidance. The study aimed to determine the prevalence of VL suppression and HIVDR among individuals who had been receiving ART for ≥ 36 months in Viet Nam.

## Methods

### Study design and sampling

In line with WHO guidance, this cross-sectional survey used a two-stage cluster design. (*5*) In the first stage, 201 clinics that had provided ART for at least three years by the end of 2013 composed the sampling frame. Clinic-level information on the number of patients starting ART and on ART for at least 36 months was not available; however, Viet Nam had reliable site-level data on the number of patients on ART. We used probability proportional to proxy size (PPPS) sampling in which the probability that a clinic was sampled is proportional to the size of the proxy patient population. The selected clinics were sampled through systematic PPPS sampling. (*5*) The number of persons receiving ART at the end of 2013 at each clinic was used as the proxy size of patients on ART at each clinic.

In the second stage, a sample of eligible patients was consecutively recruited from each of the selected clinics. The sample size for 16 representative clinics without stratification was calculated following the formula for a Wald-type confidence interval as recommended in WHO guidance. (*5*) To estimate the required sample size, the following assumptions were made: VL suppression prevalence of 70% for those receiving ART for ≥ 36 months, expected amplification failure rate at 15%, (*5*) expected proportion of individuals sampled still receiving first-line ART at 95% and expected proportion of individuals sampled on first-line ART receiving non-nucleoside reverse transcriptase inhibitor (NNRTI)-based regimens at 100%. Based on these assumptions and a desired confidence interval of ± 7%, it was estimated that a sample size of 368 persons was required, resulting in the enrolment of 23 eligible persons at each of the 16 selected clinics.

### Participant recruitment

Individuals with HIV aged 18 years or older who had been on ART for at least 36 months at the time of the clinic visit were eligible for inclusion. WHO guidance suggests conducting the survey at two treatment time points (12 ± 3 months and ≥ 48 months after initiation); (*5*) however, Viet Nam started planning the survey in late 2013, before the WHO guidance was finalized. We used the inclusion criteria listed in the draft recommendation, which was to survey adults who had received ART for ≥ 36 months.

To estimate the size of the clinic population and allow adjustments during the analysis, survey sites recorded all eligible patients who attended the clinic during the first three months of the study. At each clinic, eligible patients were enrolled consecutively until 23 patients were enrolled or until the maximum enrolment period of three months had passed, whichever came earlier. Following patient consent, blood specimens were drawn for VL measurement and genotyping. On the day of specimen collection, clinical data were also collected from the patient’s medical record by ART clinic staff, including age, sex, date of ART start and ART regimen and CD4 counts before ART initiation and the most recent results before enrolment.

### Specimen shipment and laboratory testing

Plasma specimens were tested for VL and HIVDR in two laboratories designated by WHO as national HIVDR laboratories: the National Institute for Hygiene and Epidemiology (NIHE), which tested specimens from eight outpatient clinics in the north of the country, and the Pasteur Institute in Ho Chi Minh City (PI HCMC), which tested specimens from eight outpatient clinics in the south of the country.

HIV-1 RNA viral quantification was conducted using the automated Abbott real-time HIV-1 assay (in NIHE) and the automated Roche Cobas AmpliPrep/Cobas TaqMan HIV-1 assay (in PI HCMC) with detection limits of 20 copies/mL. HIVDR genotypic test was conducted on the pol gene with the ABI 3130XL system using the Big-Dye Terminator v3.1 Cycle Sequencing Kit (Applied Biosystem, California, USA). HIVDR was interpreted using the HIValg Program on the Stanford University HIV Drug Resistance Database web site. (*6*) HIVDR was defined as low-level, intermediate or high-level resistance to one or more of the following drugs: nevirapine (NVP), efavirenz (EFV), any nucleotide reverse transcriptase inhibitors NtRTI, atazanavir (ATV), darunavir (DRV) or lopinavir (LPV). NNRTI resistance was defined as resistance to NVP or EFV, NRTI resistance was defined as resistance to any NtRTI, including abacavir (ABC), zidovudine (ZDV), emtricitabine (FTC), lamivudine (3TC), tenofovir (TDF), stavudine (D4T) and didanosine (DDI). Protease inhibitor (PI) resistance was defined as resistance to ATV, DRV or LPV. Estimates were weighted for study design.

### Data entry and statistical analysis

Data were entered using Epi Data 3.0 (EpiData Software, Odense, Denmark) and statistical analysis was performed using STATA version 11 (STATA Corp., Texas, USA). Standard descriptive statistics were calculated for categorical and continuous variables. Data analysis for prevalence of VL suppression was conducted in STATA using the survey (svy) suite of commands. Data were weighted by clinic size (i.e. the number of eligible patients screened at a clinic during the three months after the survey start date, the number of patients with VL suppression and the number of individuals with sequences genotyped). (*5*) A 95% confidence interval was calculated using a standard Wald formula or by a logit transformation. The FASTA files were submitted to the Stanford University HIV Drug Resistance Database for interpretation. (*7*) A detailed description of the technical data analysis has been described elsewhere. (*5*)

### Ethics and permissions

The study protocol was reviewed and approved by the Institutional Review Board of Hanoi University of Public Health, Hanoi, Viet Nam (Approval no: 210/2014/YTCC-HD3).

## Results

### Characteristics of study participants

During the enrolment period, a total of 6920 patients were screened at 16 sampled clinics, from which 368 eligible patients were recruited. Three patients were excluded because the duration of ART was less than 36 months; therefore, 365 persons were included in the final analysis. Their baseline characteristics are summarized in **Table 1**. The study-design-weighted mean age was 38.2 years (95% confidence interval [CI]: 37.0–39.4) and 68.4% were males. At the time of study enrolment, 93.7% of the participants were on a first-line NNRTI-based regimen, 77.8% (273/351) had advanced HIV infection (CD4 < 100 cells/ml) and the mean duration on ART was 75.5 months (95% CI: 69.0–81.9). Of the 365 patients, 55.9% (204) were on a ZDV-containing regimen, 54% (197) on a NVP-containing regimen, 40.3% (147) on an EFV-containing regimen and 8.4% (140) on a TDF-containing regimen. The adjusted proportions are presented in **Table 1**.

**Table 1:** Characteristics of individuals on current ART regimen for at least 36 months (*n* = 365)

-	n	Proportion^a^ % (95% CI)
**Gender**		
Women	118	31.6 (25.9–37.9)
Men	247	68.4 (62.1–74.1)
**Mean^b^ age (95% CI), years**	-	38.2 (37.0–39.4)
≤ 25 years	1	< 0.5
> 25 years	364	99.6 (97.1–100)
Individuals on first-line ART	345	93.8 (88.3–96.8)
Individuals on NNRTI-based first-line ART	344	93.7 (88.3–96.8)
Individuals on PI-based second-line ART	20	6.2 (3.2–11.6)
**Current ART**	-	-
TDF + XTC + EFV	93	25.6 (18.5–34.3)
TDF + XTC + NVP	47	12.3 (7.7–19.1)
TDF-based regimen	158	43.3 (33.4–53.8)
ZDV + XTC + EFV	54	13.7 (9.6–19.2)
ZDV + XTC + NVP	148	41.4 (32.9–50.5)
ZDV-based regimen	204	55.4 (45.1–65.3)
EFV-based regimen	147	39.3 (32.3–46.8)
NVP-based regimen	197	54.5 (47.5–61.3)
PI-based regimen (all LPV based)	20	6.2 (3.2–11.6)
Other	3	0.8 (0.2–2.9)
Mean^b^ time on ART (95% CI), months	-	75.5 (69.0–81.9)

^a^ Study-design-weighted proportion and 95% confidence interval

^b^ Study-design-weighted mean and 95% confidence interval

ART = antiretroviral therapy, NNRTI = non-nucleoside reverse transcriptase inhibitor, PI = protease inhibitor, TDF = tenofovir, XTC = either lamivudine or emtricitabine, EFV = efavirenz, NVP = nevirapine, ZDV = zidovudine.

### Viral load suppression

Among the 365 participants with VL testing, 345 (95.1%) achieved VL suppression (defined as VL < 1000 copies/mL). The prevalence of VL suppression among individuals on first-line ART was 94.8% (95% CI: 92.1–96.6%) (**Table 2**).

**Table 2:** Prevalence of VL suppression (< 1000 copies/mL) for individuals on ART for at least 36 months

-	n	Prevalence^a^ % (95% CI)
VL suppression among individuals on ART	345	95.1 (92.3–96.9)
VL suppression among individuals on first-line ART	325	94.8 (92.1–96.6)
VL suppression among individuals on NNRTI-based first-line ART	325	94.9 (92.1–96.7)
VL suppression among individuals on second-line ART	20	100%
VL suppression among individuals on LVP-based regimen	20	100%
VL suppression among individuals on ZDV-based regimen	192	94.7 (90.6–97.0)
VL failure among individuals on ZDV-based regimen	12	5.3 (3.0–9.4)
VL suppression among women on ART	112	95.7 (89.8–98.2)
VL suppression among men on ART	233	94.9 (90.1–97.4)
VL suppression among individuals on ART aged ≤ 25 years	-	-
VL suppression among individuals on ART aged > 25 years	344	95.1 (92.3–96.9)

^a^ Estimates were weighted for study design (see methods section).

VL = viral load.

### HIV drug resistance

The study-design-weighted prevalence of any HIV drug resistance among patients on ART was 4.6% (95% CI: 0.28–0.75) and among persons with VL > 1000 copies/ml was 94.7% (95% CI: 64.1–99.4%) (**Table 3**). Of the 20 (14.5%) persons with a detectable VL, 19 carried a virus with mutations associated with HIVDR (five persons with VL between 1000 and 5000 copies/mL and 14 persons with VL > 5000 copies/mL).

**Table 3:** Prevalence of HIVDR among individuals on ART for at least 36 months

-	n/N	Prevalence^a^ % (95% CI)
**HIVDR among individuals on ART with VL ≥ 1 000 copies/mL**		
Any	19/20	94.8 (64.4–99.5)
NNRTI	18/20	87.0 (53.6–97.5)
NRTI	18/20	87.7 (55.4–97.6)
PI	0/20	-
NNRTI+NRTI	17/20	79.9 (46.6–94.8)
**HIVDR among individuals on first-line ART with VL ≥ 1 000 copies/mL**		
Any	19/20	94.8 (64.4–99.5)
NNRTI	18/20	87.0 (53.6–97.5)
NRTI	18/20	87.7 (55.4–97.6)
PI	0/20	-
NNRTI+NRTI	17/20	79.9 (46.6–94.8)
**HIVDR among individuals on NNRTI first-line with VL ≥ 1 000 copies/mL**		
Any	18/19	94.7 (64.1–99.4)
NNRTI	17/19	86.8 (53.3–97.5)
NRTI	17/19	87.5 (55.1–97.6)
PI	0/19	-
NNRTI+NRTI	16/19	79.7 (46.1–94.7)
**HIVDR among individuals on ART**		
Any	19/365	4.6 (2.8–7.5)
NNRTI	18/365	4.2 (2.4–7.4)
NRTI	18/365	4.3 (2.4–7.4)
PI	0/365	-
NNRTI+NRTI	17/365	3.9 (2.0–7.3)

^a^ Estimates were weighted for study design (see method section). Any HIVDR is defined as low-level, intermediate or high-level resistance (according to the Stanford HIVdb) with respect to one or more of the following drugs: NVP, EFV, any N(t)RTI, ATV, DRV or LPV; NNRTI resistance is defined as resistance to NVP or EFV; NRTI resistance is defined as resistance to any N(t)RTI; and PI resistance is defined as resistance to ATV, DRV or LPV. Estimates were weighted for study design (see methods section).

ART = antiretroviral therapy, VL = viral load, NNRTI = non-nucleoside reverse transcriptase inhibitor, NRTI = nucleoside reverse transcriptase inhibitors, PI = protease inhibitor.

**Fig. 1:**
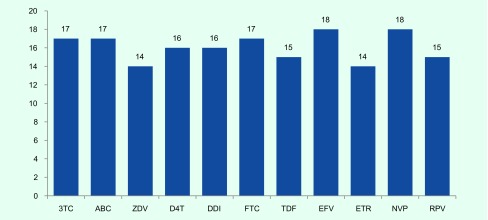
Frequency of mutations conferring resistance to NRTIs

All the detected mutations were associated with resistance to reverse transcriptase inhibitors, and no major mutations associated with resistance to PI were found (**Fig. 1**). In the 19 cases with drug resistance mutations, one case (5.3%) had mutations associated with only NRTI resistance, another one (5.3%) had mutations associated with only NNRTI resistance, and the remaining 17 cases (89.4%) had mutations associated with both NRTI and NNRTI resistance. In cases with drug-resistance mutations to both NRTI and NNRTI, the mean viral load was 45 556 copies/mL (95% CI: 16 603–74 509).

3TC = lamivudine, ABC = abacavir, ZDV = zidovudine, D4T = stavudine, DDI = didanosine, FTC = emtricitabine, TDF = tenofovir, EFV = efavirenz, ETR = etravirine; NVP = nevirapine, RPV = rilpivirine.

Among the 20 patients with a detectable VL, the most common NNRTI mutations were Y181C (10/20, 50%), K103N (7/20, 35%), V106I (7/20, 35%) and G190A (7/20, 35%). For NRTI resistance, the most common resistance mutations were M184V (16/20, 80%); V75M (5/20, 25%); and thymidine analogue mutations (TAMs), consisting of T215F/I/Y (12/20, 60%), K219E/Q (9/20, 45%), K70R (9/20, 45%), D67N (8/20, 40%), M41L (7/20, 35%) and L210W (4/20, 20%). There were 45% (9/20) of patients harbouring viruses with three or more TAMs Of the 20 patients failing ART, 75% (15) had mutations that predict resistance to tenofovir, while 85% (17) and 70% (14) had mutations that predict resistance to either lamivudine (3TC) or emtricitabine (FTC) and ZDV, respectively (**Fig. 1**). Prevalence of NNRTI resistance ranged from 70% (14/20) for etravirine (ETR) to 90% (18/20) for EFV and/or NVP (**Fig. 1**).

### Association between CD4 count and HIVDR mutations

**Table 4** shows the results of bivariate analysis and multivariate analysis assessing the relationship between CD4 counts with the presence of HIVDR mutations. Bivariate analysis showed that CD4 counts < 100 cells/mm3 or between 100 and 350 cells/µl were associated with HIVDR mutations. In multivariate analysis, these two conditions were independently associated with the presence of HIVDR mutations: adjusted odds ratio (aOR) = 98.3 (95% CI: 10.9–888.2) for CD4 < 100 cells/mm^3^ and aOR = 11.4 (95% CI: 2.51–51.9) for CD4 between 100 and 350 cells/µl.

**Table 4:** Correlates of HIVDR mutation (any mutation)

Variables	N	Any mutation	Crude OR (95% CI)	P-values	Adjusted OR (95% CI)^a^	P-values
**Provinces**						
Hanoi	70	4 (5.7%)	0.776 (0.199–3.019)	0.714	-	-
Other provinces in the north	111	7 (6.3%)	0.862 (0.262–2.829)	0.806	-	-
Ho Chi Minh City	115	3 (2.6%)	0.343 (0.079–1.482)	0.152	-	-
Other provinces in the south	69	5 (7.2%)	1	-	-	-
**Administration level**						
District level	184	8 (4.3%)	0.702 (0.276–1.789)	0.459	-	-
National or provincial level	181	11 (6.1%)	1	-	-	-
**Years, median (IQR)**	-	37 (33–42)	-	-	-	-
**Age group**						
Under 35 years old	148	8 (5.4%)	1.07 (0.42–2.728)	0.887	1	-
From 35 years old and above	217	11 (5.1%)	1	-	1.334 (0.393–4.526)	0.644
**Sex**						
Male	274	13 (5.3%)	1.037 (0.384–2.8)	0.943	0.363 (0.8–1.643)	0.188
Female	118	6 (5.1%)	1	-	1	-
**Months from ART to sampling**	-	65 (57–85)	-	-	-	-
**ART duration**						
From 36 months to less than 60 months	108	7 (6.5%)	1.571 (0.512–4.817)	0.429	1	-
From 60 months to less than 84 months	115	6 (5.2%)	1.248 (0.391–3.977)	0.708	1.383 (0.359–5.324)	0.63
From 84 months and above	142	6 (4.2%)	1	-	0.818 (0.189–3.55)	0.789
**Self-reported mode of infection**						
Injection drug use	141	9 (6.4%)	1.455 (0.562–3.762)	0.440	1.538 (0.420–5.631)	0.516
Heterosexual	201	9 (4.5%)	1	-	1	-
MSM	1	0 (0.0%)	-	-		
**WHO stage before ART initiation**						
1	46	1 (2.2%)	1	-	-	-
2	50	2 (4.0%)	1.875 (0.164–21.397)	0.613	1.538 (0.106–22.245)	0.752
3	152	3 (2.0%)	0.906 (0.092–8.926)	0.933	1.18 (0.106–13.094)	0.893
4	108	13 (12.0%)	6.158 (0.781–48.54)	0.084	7.265 (0.746–70.748)	0.088
**History of ARV exposure before ART start**						
Yes	24	1 (4.2%)	0.807 (0.102–6.36)	0.839	-	-
No	313	16 (5.1%)	1	-	-	-
**TB treatment history after registration at OPC**						
Yes	58	2 (3.4%)	0.578 (0.13–2.571)	0.471	-	-
No	292	17 (5.8%)	1	-	-	-
**CD4 count before ART, median (IQR) (cells/mm3)**		124 (43–179)	-	-	-	-
**Most recent CD4 count, median (IQR) (cells/mm3)**		254 (115–320)	-	-	-	-
**CD4 count before ART start (cells/mm3)**						
< 100	166	8 (4.8%)	0.686 (0.268–1.752)	0.431	-	-
100–350	160	11 (6.9%)	1	-	-	-
> 350	25	0	-	-	-	-
**Most recent CD4 count (cells/mm3)**						
< 100	8	4 (50.0%)	77.3 (12.9–465)	0.000	98.304 (10.88–888.183)	< 0.001
100–350	119	12 (10.1%)	8.67 (2.40–31.4)	0.001	11.413 (2.509–51.921)	0.002
> 350	235	3 (1.3%)	1	-	1	-

^a^ Adjusted for age group, sex, ART duration, self-reported mode of HIV infection, pre-ART WHO clinical stage, history of ARV exposure before ART start and the most current CD4 count.

IQR = interquartile range, TB = tuberculosis, ARV = antiretroviral, OPC = HIV outpatient clinic, ART = antiretroviral therapy, MSM = men who have sex with men, OR = odds ratio, CI = confidence interval

## Discussion

This study was the first survey of ADR in Viet Nam following the WHO guidance for ADR surveillance released in 2014, (*5*) and Viet Nam was one of the first countries in the world to adopt the new WHO ADR survey protocol. (*8*) The new WHO protocol is aimed at obtaining nationally representative estimates of VL suppression and ADR using a cross-sectional design in contrast to the previous prospective cohort method that focused on sentinel ART clinics. (*4*) Our survey proved that the new cross-sectional WHO approach is feasible to implement in Viet Nam and is able to generate important information that could be used to optimize ART programmes.

In our survey, the level of VL suppression was one of the highest and the ADR level the lowest among the four countries that have reported the results of an ADR survey using the new WHO protocol. (*8*)

In the past decade, various surveys of HIVDR were conducted in Viet Nam that reported levels of transmitted HIV drug resistance (TDR), pre-treatment HIV drug resistance (PDR) and ADR. In a study among 70 newly diagnosed HIV-positive clients aged 18–24 years in Hanoi in 2006, the prevalence of TDR was at a low level (< 5% to all drugs), (*9*) while a moderate resistance prevalence (5–15%) of TDR to NNRTIs was observed among similar clients (aged 18–21 years at voluntary counselling and testing sites) in Ho Chi Minh City in 2007–2008. (*10*) A five-year study (2008–2012) among 1426 ART-naïve patients in a single hospital in southern Viet Nam indicated that the annual prevalence of TDR remained low to moderate (2.4–5.48%). (*11*) A prospective cohort study of ADR conducted between 2009 and 2012 at four treatment clinics (two clinics in Ho Chi Minh City and two clinics in northern Viet Nam) showed that PDR to the drugs used in the first-line ART regimen was 2.7% (95% CI: 1.6–4.4%) (13/490 participants). (*12*) This study also showed 91.3% (CI: 95%: 87.0–97.9%) of patients achieved VL suppression at 12 months after ART initiation, 2.9% of patients had developed an HIVDR to NNRTIs or NRTIs at 12 months after ART initiation and no patients had developed detectable PI resistance. (*13*) A cross-sectional study at three clinics in Ho Chi Minh City in 2009–2011 reported the level of ADR among those receiving ART for 12 ± 2 months and 24 ± 2 months were 22/296 patients (7.4%) and 25/300 patients (8.3%), respectively. (*14*)

These previous studies in Viet Nam did not use a nationally representative sample and thus were limited by potential site selection bias. At the same time, these results suggest that HIVDR has been at a low level in Viet Nam, and the results of the present survey are in line with these previous findings. However, it should also be noted that TDR (*10*) and ADR (*14*) surveys conducted in Ho Chi Minh City report somewhat higher (moderate) levels of HIVDR compared to the rest of the country. While it is important to generate nationally representative estimates, future studies may also require stratification of sampling to understand potential geographical differences.

The present survey also showed that over 95% of people with HIV who are receiving ART for more than three years have suppressed VL (< 1000 copies/ml). This study result is also in line with other studies showing that Viet Nam’s programmes are achieving a high level of VL suppression (< 1000 copies/ml) at 12 months as reported from cohort studies (*13*, *15*) and a cross-sectional survey. (*16*)

Our study found a strong association between VL and drug-resistance mutations among patients receiving ART for at least 36 months. HIVDR mutations to NNRTI were detected in 19 out of 20 (95%) patients with VL > 1000 copies/ml supporting the notion that a prompt switch to second line therapies is needed in people with detectable virus despite treatment. Because HIVDR is associated with a low level of adherence (more importantly with NNRTI resistance emergence) (*17*) in settings with low levels of NNRTI resistance on ART, (*8*) strategies to improve ART adherence are critical to prevent widespread reliance on alternate treatment regimens.

In the above-mentioned study among Vietnamese adults initiating first-line ART, the percentages of patients with virologic failure (VL > 1000 copies/ml) were 11.5% (95% CI: 7.8–15.1) at 10–14 months and 10.3% (95% CI: 6.9–13.8) at 22–24 months. The percentages of patients with detectable VL that had drug-resistance mutations were 75.9% at 10–14 months and 86.2% at 22–24 months. (*14*) It is possible the presence of drug-resistance mutations is correlated with time on ART. (*18*) Following the WHO recommendation to conduct the ADR survey at two time points would enable comparisons of the level of VL suppression and patterns of HIVDR between patients found to be failing ART in the short- and long-term.

The 2010 pre-treatment HIVDR study found that the major drug-resistance mutations to the available first-line ARTs were K103N, Y181C, Y188C, G190A (NNRTI resistance), V75M and M184V (NRTI resistance). (*12*) Due to the limitations of cross-sectional design, it was not possible in our current study to determine whether the HIVDR mutations stemmed from insufficient drug pressure during ART treatment or had pre-existed from transmitted resistance before ART initiation.

The mutation pattern in our study was similar to the results of an ADR survey at three clinics in Ho Chi Minh City in 2009–2010. Among the 22 patients with HIVDR mutations at 12 months, resistance to NRTIs, NNRTIs and to both classes were reported as 4.5%, 9% and 90%, respectively. At 24 months following ART initiation, there were 25 cases with HIVDR mutations: 96% were resistant to both NRTIs and NNRTIs, 0% were resistant to NRTIs alone and 4% were resistant to NNRTIs alone. (*14*)

In conclusion, this is the first survey to describe nationally representative levels of VL suppression and ADR in adults receiving ART for at least 36 months in Viet Nam. The survey found high levels of VL suppression, low levels of ADR among people on ART and high levels of HIVDR among people failing ART, suggesting that Viet Nam had successfully managed its programme quality to maintain ADR at a low level at the time the survey was conducted in 2014.
